# Viral RNA-dependent RNA polymerase mutants display an altered mutation spectrum resulting in attenuation in both mosquito and vertebrate hosts

**DOI:** 10.1371/journal.ppat.1007610

**Published:** 2019-04-04

**Authors:** K. Lane Warmbrod, Edward I. Patterson, Tiffany F. Kautz, Adam Stanton, Dedeke Rockx-Brouwer, Birte K. Kalveram, Kamil Khanipov, Saravanan Thangamani, Yuriy Fofanov, Naomi L. Forrester

**Affiliations:** 1 Department of Pathology, University of Texas Medical Branch, Galveston, Texas, United States of America; 2 Department of Microbiology and Immunology, University of Texas Medical Branch, Galveston, Texas, United States of America; 3 School of Computing and Mathematics, University of Keele, Keele, United Kingdom; 4 Sealy Center for Structural Biology and Molecular Biophysics, Department of Pharmacology and Toxicology, University of Texas Medical Branch, Galveston, Texas, United States of America; Institut Pasteur, FRANCE

## Abstract

The presence of bottlenecks in the transmission cycle of many RNA viruses leads to a severe reduction of number of virus particles and this occurs multiple times throughout the viral transmission cycle. Viral replication is then necessary for regeneration of a diverse mutant swarm. It is now understood that any perturbation of the mutation frequency either by increasing or decreasing the accumulation of mutations in an RNA virus results in attenuation of the virus. To determine if altering the rate at which a virus accumulates mutations decreases the probability of a successful virus infection due to issues traversing host bottlenecks, a series of mutations in the RNA-dependent RNA polymerase of Venezuelan equine encephalitis virus (VEEV), strain 68U201, were tested for mutation rate changes. All RdRp mutants were attenuated in both the mosquito and vertebrate hosts, while showing no attenuation during *in vitro* infections. The rescued viruses containing these mutations showed some evidence of change in fidelity, but the phenotype was not sustained following passaging. However, these mutants did exhibit changes in the frequency of specific types of mutations. Using a model of mutation production, these changes were shown to decrease the number of stop codons generated during virus replication. This suggests that the observed mutant attenuation *in vivo* may be due to an increase in the number of unfit genomes, which may be normally selected against by the accumulation of stop codons. Lastly, the ability of these attenuated viruses to transition through a bottleneck *in vivo* was measured using marked virus clones. The attenuated viruses showed an overall reduction in the number of marked clones for both the mosquito and vertebrate hosts, as well as a reduced ability to overcome the known bottlenecks in the mosquito. This study demonstrates that any perturbation of the optimal mutation frequency whether through changes in fidelity or by alterations in the mutation frequency of specific nucleotides, has significant deleterious effects on the virus, especially in the presence of host bottlenecks.

## Author summary

RNA viruses replicate with a high mutation rate, giving them the ability to generate mutations that might be beneficial under different selection pressures. Any perturbation of the accumulation of mutations has been shown to result in severe attenuation of the virus. However, the mechanism of this attenuation is still unclear. Mosquito-borne viruses, which are mostly RNA viruses, undergo repeated bottleneck events during the transmission cycle of the virus. Using a model system of Venezuelan equine encephalitis virus (VEEV) we investigated the effect of perturbing the rate of mutations in both a mosquito and vertebrate host. We show that any perturbation of the mutation frequency, either by increasing or decreasing the overall mutation frequency as well as altering the specific frequency of mutations results in attenuation. Our results also suggest that RNA viruses may use stop codon accumulation as a means of purifying out less-fit genomes by targeting them for destruction. These changes in the genome result in viruses unable to overcome the bottlenecks in the mosquito vector and to a lesser extent in the vertebrate host. Overall, our study shows that any change in the accumulation of mutations from wild-type increases the likelihood of an unsuccessful transmission cycle.

## Introduction

RNA viruses comprise a diverse group of viruses, which exhibit high genome plasticity due to a high rate of mutation. This results in the generation of a closely related cloud of viral variants known as a ‘quasispecies’ or viral swarm [1]. The generation of this viral swarm is a result of the viral RNA-dependent RNA polymerase (RdRp), which does not possess a proof-reading function. Rather than producing perfect copies of the genome, the RdRp randomly incorporates incorrect nucleotide bases along the genome, generating a cloud of viral genomes that contain one or two mutations in each genome [2]. These variants are believed to collectively contribute to an interactive population that together create the viral phenotype. This population of variants is subject to selection as a whole, rather than selection acting on individual variants. The presence of this diversity is thought to be an advantage for viral transmission and invasion of host tissues [3]. One benefit of adopting a high mutation strategy is that this allows viruses to produce multiple advantageous mutations. The virus is therefore able to infect and replicate in multiple tissue types, each with their own different selective pressures. For arboviruses (arthropod-borne viruses) creating a diverse quasispecies is hypothesized to be of utmost importance, as two diverse species, a vertebrate host and invertebrate vector, must become infected to complete a transmission cycle, and recent work has demonstrated that diversity is important in successful completion of a transmission cycle [4–7].

Bottlenecks are present in all transmission cycles as viruses move from one tissue to another. Studies using marked clones have shown that bottlenecks are more common than were previously thought [5, 6, 8], and, at least in some systems, conform to the stochastic nature of bottlenecks so that the virus that traverses the bottleneck is selected at random. What is still unclear is how important bottlenecks are in altering viral diversity, and the robustness of viruses with altered diversity to traverse bottlenecks. Recent studies using wild-type viruses have shown that the virus is able to recover diversity within four days of experiencing the bottleneck, such that the diversity in the next tissue is similar to that in the original tissue [7]. However, successful recovery following the bottleneck is hypothesized to be associated with the successful generation of minority variants.

Virologists have come to understand that any perturbation of virus diversity has adverse consequences for the virus population as a whole. Almost all viruses that have been demonstrated to have either higher or lower diversity are attenuated compared to the wild-type *in vivo*, but replicate similarly to wild-type *in vitro* [9–14]. These viruses are collectively known as fidelity variants, as they either incorporate a higher or lower number of mutations than the wild-type. High-fidelity variants incorporate lower numbers of mutations, where-as low-fidelity mutants incorporate more mutations than the wild-type virus. Generally, the changes in fidelity are thought to be due to two different, but related mechanisms; the speed of the polymerase or mutations either distal or proximal to the active site that result in altered fidelity of the RdRp [15].

Previous work with Venezuelan equine encephalitis virus (VEEV) in the mosquito vector *Culex taeniopus* has demonstrated the presence of bottlenecks during normal transmission and infection of the mosquito vector. VEEV is a New World encephalitic alphavirus endemic to parts of Central and South America, and is transmitted between mosquitoes and small rodents. Although often misdiagnosed as dengue fever due to the constant circulation in the same geographic area, VEEV is thought to cause tens of thousands of human infections annually [16]. Importantly, VEEV is considered a potential threat to biosecurity due to its high infectivity via the aerosol route. To determine the how perturbing the mutation spectrum of RNA viruses affects its ability to move through bottlenecks, a series of mutations in the RdRp of the VEEV strain IE 68U201 were created and validated for their changes in fidelity and alterations in mutation frequency. These mutants were then tested to assess their ability to traverse bottlenecks in the vertebrate host, modelled using a wild-type mouse, and the mosquito vector (*Cx*. *taeniopus*).

## Results

### Generation of RdRp mutants

**Fig 1:**
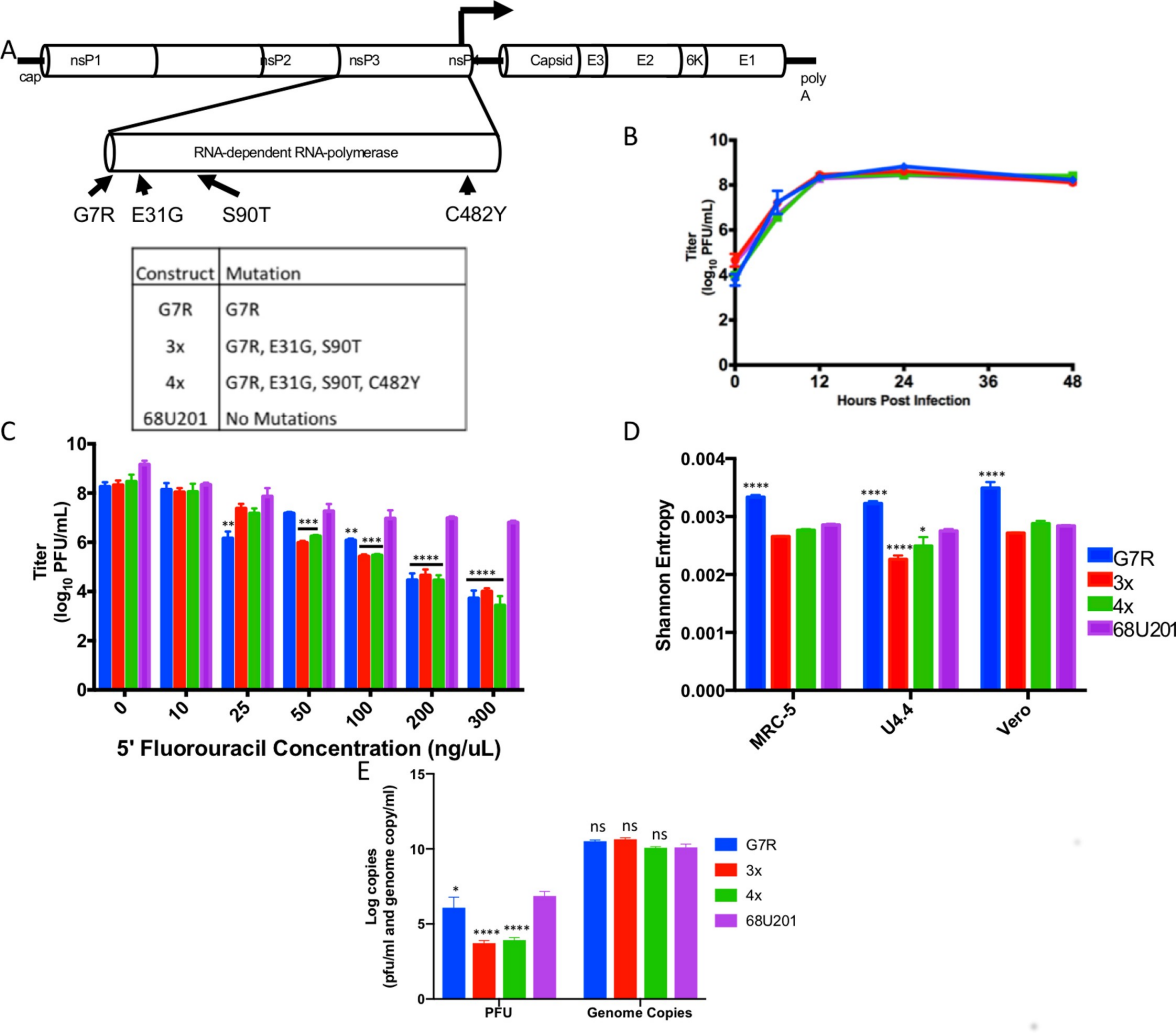
The effect of RdRp mutations on 68U201. A). The location of the mutations included in each RdRp mutant. B). Replication kinetics were assessed using a growth curve (MOI 10) of the RdRp mutants and the wild-type virus 68U201 in Vero cells performed in triplicate. No significant difference was determined by one-way ANOVA. C). Sensitivity of mutants and wild type virus to 5FU. Significance tested using two-way ANOVA. D). Shannon entropy of the RdRp mutants following one passage in MRC-5, U4.4 and Vero cells performed in triplicate. Statistical significance was determined by ANOVA p = 0.006, post hoc correction was performed using Tukey’s multiple comparison test and statistically significant changes versus the wild-type are depicted on the figure. E) The number of genome copies versus the number of plaque forming units for each of the RdRp mutants. The wild-type had a specific infectivity of 6x10-4, whereas the G7R mutant has a SI of 9x10-6, and the 3x and 4x mutants have a SI of 1x 10–7 and 7x10-7, respectively. Statistical significance was determined by ANOVA p<0.0001, post hoc correction was performed using Tukey’s multiple comparison test and statistically significant changes versus the wild-type are depicted on the figure. **** p<0.0001, *** p<0.001, ** p<0.01, * p< 0.05.

Previous work with the vaccine strain of VEEV, TC-83, demonstrated that three mutations in the RdRp collectively contributed to a decrease in virus fidelity, resulting in attenuation in a vertebrate model [11]. To determine the effects of these mutations in a different virus subtype, the mutations identified in TC-83 plus one identified in chikungunya virus (CHIKV) [10] were inserted in the 68U201 backbone and rescued, generating three RdRp mutants (Fig 1A). The first RdRp mutant contained a single nucleotide mutation producing the single G7R amino acid change in the RdRp. The second RdRp mutant, the 3x mutant, contained three amino acid mutations in the RdRp, including G7R, E31G, and S90T. The final RdRp mutant, 4x mutant, contained the following amino acid mutations in the RdRp: G7R, E31G, S90T, and C482Y. To verify that the inserted mutations did not change the replication kinetics of the viruses, growth curves at a multiplicity of infection (MOI) of 10 were conducted for all mutants and the wild-type virus 68U201 (Fig 1B). All mutants replicated to similar titers and there were no statistical differences between mutants and wild-type as determined by one-way ANOVA.

Resistance to nucleoside analogues has been one of the tools for determining the presence of a fidelity mutation, as high fidelity viruses are less likely to incorporate mutagens, while low-fidelity viruses should be more susceptible [14, 17, 18]. Thus, to determine if these mutations confer resistance or increased sensitivity to RNA mutagens, each RdRp mutant was tested for resistance to 5’fluorouracil (5FU) at a range of concentrations (Fig 1C). All three RdRp mutants showed significantly lowered titers compared to wild-type at concentrations of 100 ng/μl 5FU or higher as determined by two-way ANOVA.

To determine if these inserted mutations altered virus fidelity, each RdRp mutant along with the wild-type virus were used to infect the vertebrate cell lines, MRC-5 and Vero, and the mosquito cell line U4.4 at an MOI of 0.1. At 24 hours post infection, the viruses were harvested and the viral RNA extracted and sequenced using Illumina sequencing to determine the amount of diversity within each virus population (Fig 1D). The G7R mutation significantly increased virus diversity compared to the wild-type strain when tested by two-way ANOVA (p<0.0001) in all three cell types. The 3x mutation showed a significant reduction in diversity compared to the wild-type strain in U4.4 cells (p<0.0001), and a non-significant reduction in Vero and MRC-5 cells. The 4x mutant showed a slight reduction in diversity, but this was only significant in U4.4. cells (p = 0.05) and not in either MRC-5 or Vero cells.

To determine the stability of the mutations and the phenotype associated with them, the wild-type and RdRp mutant viruses were passaged five times using Vero cells. Following the fifth passage, the growth kinetics and sensitivity to 5FU assays were repeated with passage 5 virus (S1 Fig). There was no significant difference in viral titer between any RdRp mutant and the wild-type virus at any time point of the growth curve or any concentration of 5FU.

To verify the mechanism of attenuation of the RdRp mutants, stocks of the electroporated viruses were titered to determine the number of viable viral particles, as well as the number of RNA genomes produced as determined by real-time RT-PCR (i.e. to determine the specific infectivity (SI) of the viruses). All four viruses produced equivalent amounts of genome copies to the wild-type, which shows that the rate of genome replication was not altered as a result of the mutations (Fig 1E). However, the titer of the RdRp mutants was significantly decreased as compared to the wild-type virus. Thus, the SI of the mutants is reduced compared to the wild-type. The wild-type had a SI of 6x10^-4^, whereas the G7R mutant has a SI of 9x10^-6^, and the 3x and 4x mutants have a SI of 1x 10^−7^ and 7x10^-7^, respectively.

### Mutation changes associated with the altered phenotypes

**Fig 2:**
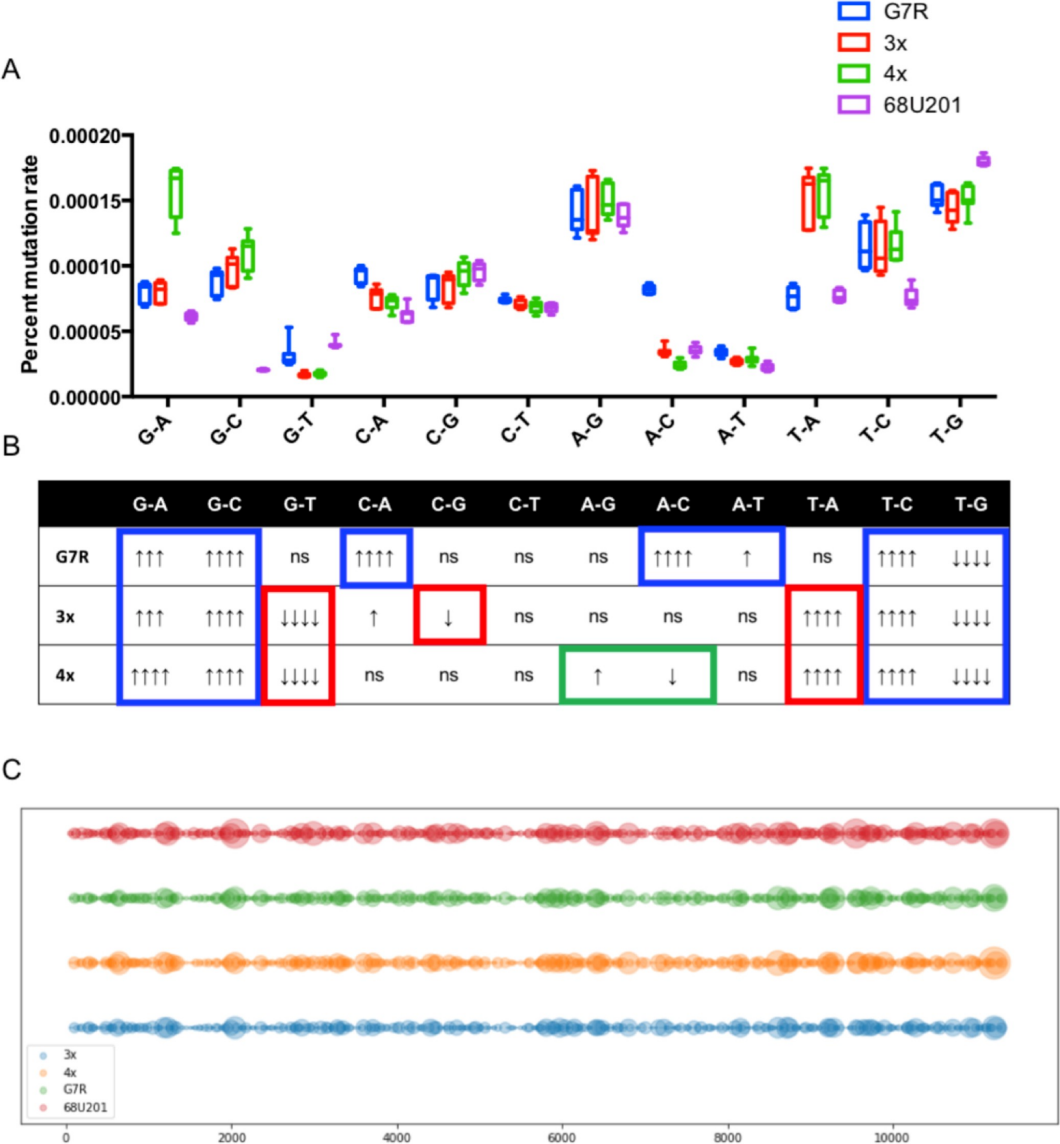
Changes in mutation preference following mutations in the RdRp. A). The changes in the proportion of different mutation types in the different RdRp mutants after passage 1 in MRC-5, U4.4 and Vero cells. B). Table showing the mutation types and the statistical changes from wild-type. Statistical significance was determined by ANOVA p<0.0001, post hoc correction performed using Dunnett’s multiple comparisons test. Arrows indicate change in frequency, and significance. Blue boxes indicate changes associated with the G7R mutation, red boxes indicate changes associated with the G7R, E31G and S90T mutations, and green boxes indicate changes associated with the G7R, E31G, S90T and C482Y mutations. ↑↑↑↑ p< 0.0001, ↑↑↑ p<0.001, ↑↑ p<0.01, ↑ p<0.05. C). The number of stop codons produced when the mutation frequencies are altered (for mutation changes see Table 1). Showing the numbers of stop codons, larger circles correspond to an increased number of stop codons present in the genome. The X-axis shows the nucleotide position of the mutations showing stop codons.

To test if the apparent changes in entropy were the result of particular mutational biases, the propensity for specific base changes was determined for the MRC-5, Vero and U.4.4 cells individually (S2 Fig) and combined (Fig 2). There were significant differences associated with specific base changes as determined by two-way ANOVA and each change from the wild-type 68U201 is shown in Fig 2 for both individual cell types and when all cell types were combined. The G7R mutation resulted in a significant increase in the number of G-A, G-C, C-A, A-C, A-T and T-C mutations, while decreasing the number of T-G mutations. The 3x RdRp mutant produced a significantly higher proportion of T-A mutations and significant decreases of G-T mutations. However, the addition of the two extra mutations, E31G and S90T, reduced the effect of the C-G and the A-C changes so that no significant change in base changes was observed compared to that observed in the G7R mutation. The addition of the C482Y mutation in the 4x mutant resulted in an increase in the number of A-G mutations and a decrease in the number of A-C mutations, albeit at a lower significance than associated with the other RdRp mutants. As these RdRp mutants caused changes in mutation frequency that resulted in attenuation, it was hypothesized that these changes altered the number of viruses with viable genomes, i.e. increased the number of stop codons resulting in more unfit viral particles. To determine if these mutational changes resulted in an increase in truncated genomes, a model was set up to determine if these alterations would result in more or less lethal mutations. The specific base frequency changes shown in Table 1 were used to generate mutations randomly along the coding portion of each genome at a rate of 1 mutation in every 10,000 nucleotides added. One hundred billion genomes were generated, and then each *in silico* nascent genome was translated to determine the number of additional stop codons generated compared to the wild-type 68U201. Although it was expected that the number of stop codons would increase, the model indicates that the alteration in base changes actually leads to a reduced number of stop codons compared to the wild-type 68U201 virus (p<0.0001, by one-way ANOVA) (Fig 2C) (S1 Table). Translation of the data used to generate the frequencies showed the same reduction in the number of stop codons in the RdRp mutants (S2E Fig).

**Table 1:** The mutation proportions used to generate the model that determined the number of stop codons produced as a result of the mutations.

	G7R	3x	4x	68U201
**G-A**	0.07645003	0.07667533	0.13718076	0.069933
**G-C**	0.08419321	0.09463397	0.09510129	0.02328946
**G-T**	0.02957334	0.0160386	0.0152596	0.04554446
**C-A**	0.08857339	0.07334453	0.06261927	0.07154988
**C-G**	0.08100787	0.08144008	0.08215234	0.10930843
**C-T**	0.07039774	0.06803688	0.06004696	0.07756706
**A-G**	0.13299544	0.1352778	0.13136807	0.15698207
**A-C**	0.07799078	0.03321736	0.02102178	0.04063423
**A-T**	0.03250486	0.02575385	0.02522094	0.02551376
**T-A**	0.07249201	0.14717589	0.13576939	0.08778562
**T-C**	0.10876877	0.10889809	0.10137649	0.08651281
**T-G**	0.14505255	0.13950761	0.13288312	0.20537921

### Determining the attenuation of the RdRp mutants in the vertebrate host

**Fig 3:**
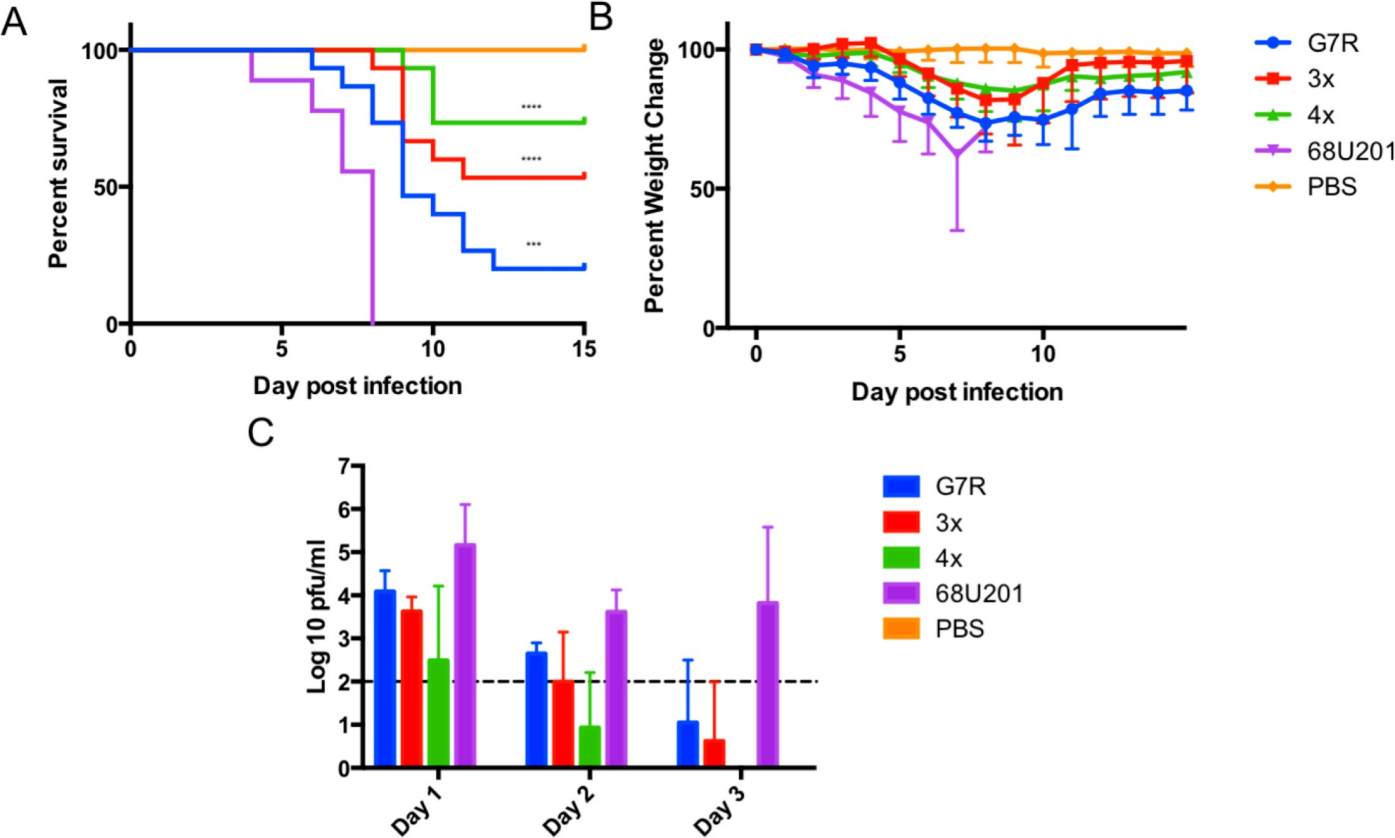
Decreased virulence of RdRp mutants in adult CD-1 mice. A) The survival of the animals following inoculation with 3log_10_ of each of the three RdRp mutants and the wild-type 68U201 strain. Statistical significance was determined by the Log-Rank (Mantel Cox test). B) The percent weight change associated with the animals, the dashed line shows the 70% weight cutoff and C) the viremia titers from days 1 to 3 post infection. Statistical significance between the RdRp mutants and wild-type, 68U201 strain, was determined by ANOVA p<0.0001, post hoc correction performed using Tukey’s multiple comparison test against the wild-type. Statistical difference for G7R, 3x and 4x was p<0.0001, dashed line indicates the LOD for the plaque assay. **** p<0.0001, *** p<0.001. Results are combined from two independent experiments.

RdRp mutant and wild-type viruses were inoculated into mice to determine the effects of the RdRp mutations on virulence. There was a reduction in mortality and increase in time to death for the RdRp mutants compared to the wild-type 68U201 in adult mice (Fig 3A). All of the RdRp mutants showed a significant reduction in mortality as measured by the Log-Rank test. This was also reflected in weight, with the wild-type virus (68U201) showing increased weight loss compared to the mutated viruses (Fig 3B). Interestingly, some animals survived even after 30% weight loss when infected with the G7R mutant. These animals did show evidence of neurological disease following a gain in weight, but animals were still gaining weight when the experiment was terminated. This is highly unusual for VEEV [19], and suggests that these animals were able to clear the virus from the brain and recover from the severe disease. Therefore, viremia titers were examined for each animal to determine the severity of the viremia compared to the wild-type 68U201. For all the RdRp mutants there was a significant drop in the amount of virus in the blood compared to the wild-type 68U201 strain with p<0.0001 by one-way ANOVA (Fig 3C).

### Determining the ability of the RdRp mutants to disseminate in arthropod vector

**Fig 4:**
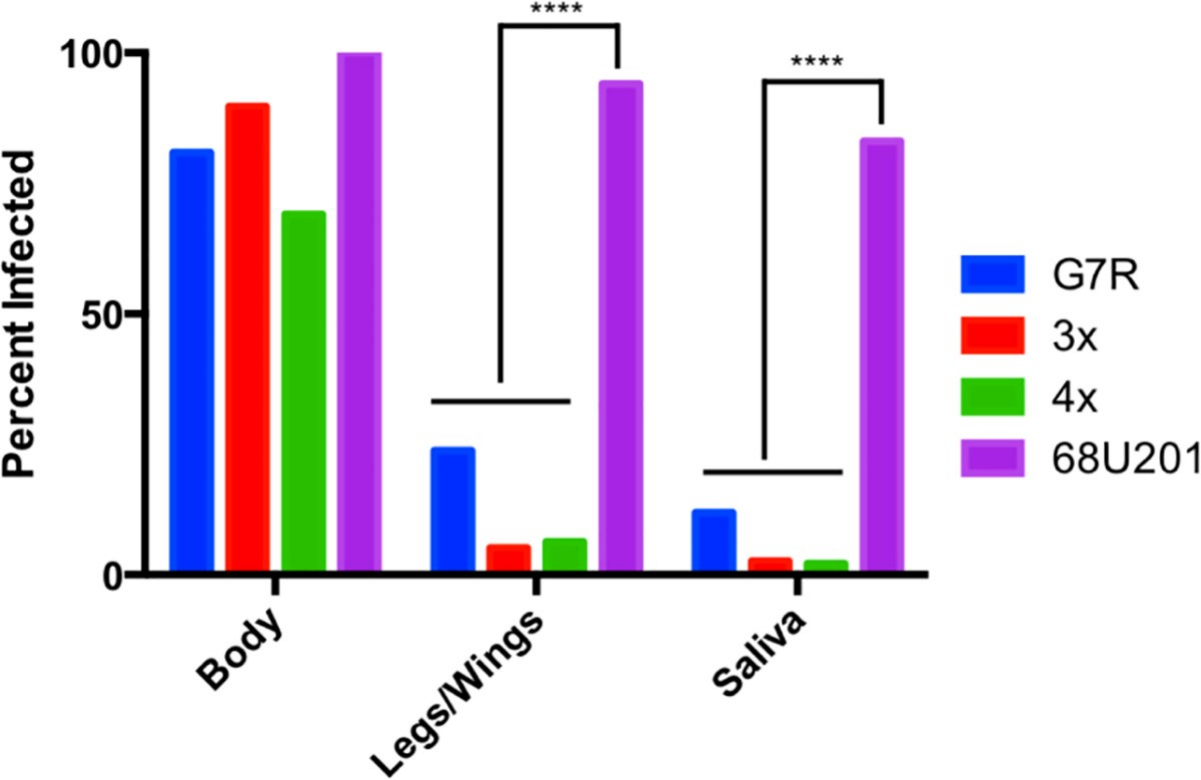
The reduction in dissemination of the altered fidelity variants in mosquitoes. Mosquitoes were harvested at days 4, 8, and 12, when 95% of wild-type mosquitoes are infected, numbers of mosquitoes in the experiment and a breakdown of the days can be found in S3 Fig. Statistical significance was determined by Kruskal-Wallis test, post hoc correction was performed by Dunn’s multiple comparison test and significant results are indicated on the graph. **** p<0.0001.

When the RdRp mutants were used to infect cohorts of *Cx*. *taeniopus* mosquitoes, there was a significant reduction in the number of disseminated infections compared to the wild-type 68U201 strain (Fig 4). The percent of mosquitoes with infections in the legs/wings and salivary glands was significantly reduced for all three RdRp mutants compared to the wildtype virus.

### The effect of altered mutation frequencies on bottlenecks in the mosquito host

To test how altered mutation frequencies affected the ability of the virus to traverse a bottleneck, a series of silent mutations were inserted into the G7R, 3x and 4x RdRp mutants, generating 8 neutral marked viruses of each RdRp mutant. These marked viruses were previously described in Forrester et al. 2012 [6], and used to determine the number of bottlenecks in the mosquito *Cx*. *taeniopus*. Mice were intravenously infected with an equal amount of each marked virus so that they exhibited an artificial viremia of approximately 10^5^ PFU/ml or 10^6^ PFU/ml, and mosquitoes were allowed to feed on the mice for 45 minutes. Mice were bled both before and after the feed to verify the final titer of the blood fed to mosquitoes. Mosquitoes were then sampled on day 1 (midguts and bodies), and days 4, 8, and 12 (bodies, legs/wings and saliva). The number of marked viruses present in each sample was determined using a real-time assay that allowed each marked virus to be identified.

**Fig 5:**
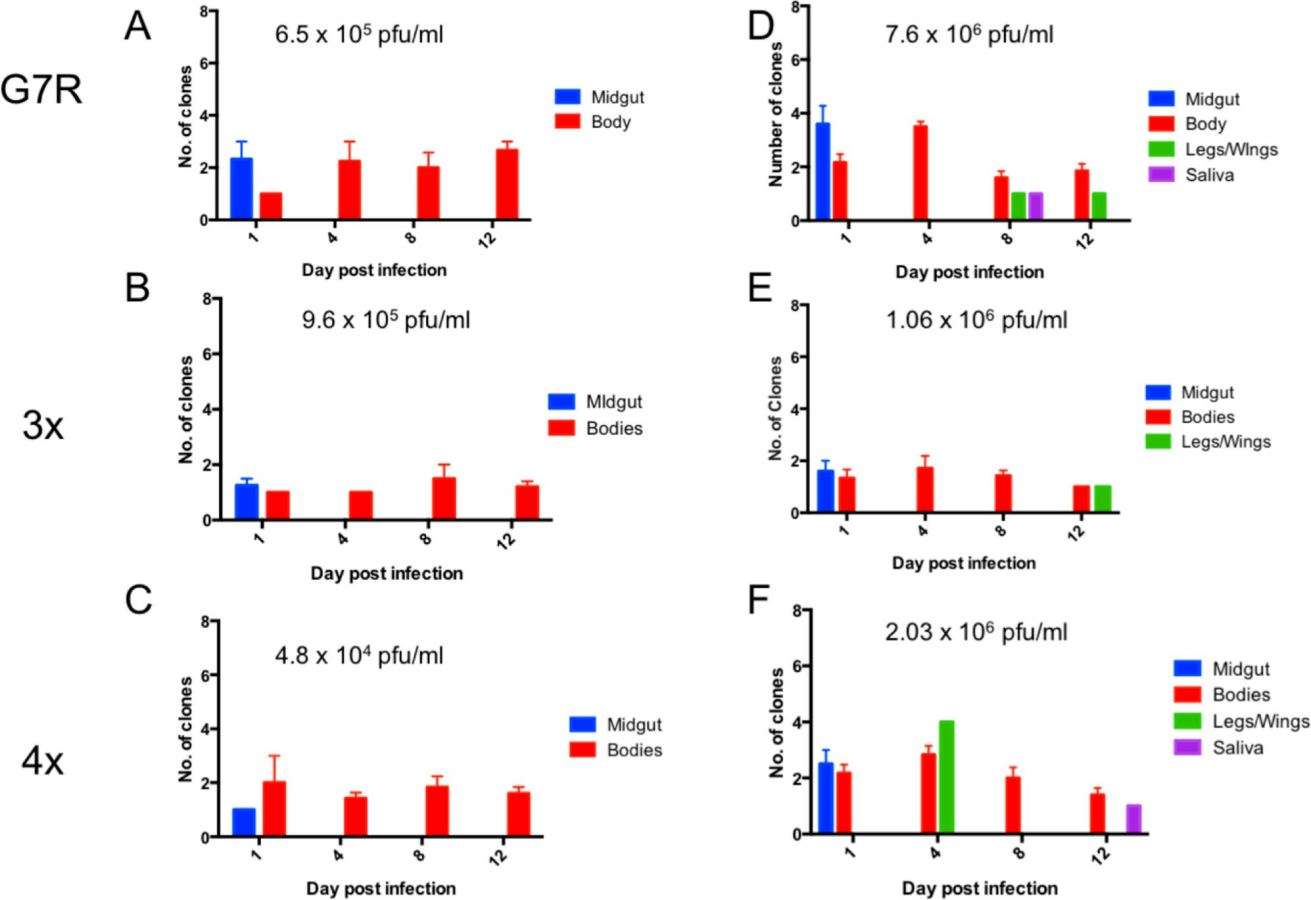
The numbers of marked viruses present in each of the mosquito compartments following infection with a mixture of 8 marked viruses. A, B & C infections with low dose for G7R, 3x and 4x RdRp mutants, respectively. D, E & F, infections with high dose for G7R, 3x and 4x RdRp mutants, respectively.

When exposed to 6.5 x 10^5^ PFU/ml of the G7R marked viruses, none of the mosquitoes had virus dissemination outside of the midgut/body (Fig 5A). These mosquitos had a mean of 3 marked viruses in the midgut and 1.667 in the bodies. However, when the mosquitoes were exposed to 7.6 x10^6^ PFU/ml of the G7R marked virus mixture, there were 3.6 marked viruses on average that initiated infection in the midguts, and 2.281 in the bodies (Fig 5D). The legs/wings showed an average of 1 marked virus per tissue. Only one mosquito was positive for virus in the saliva, and this sample also had only one marked virus.

For the 3x mutant, none of the mosquitoes showed dissemination outside the midgut/bodies when the mosquitoes were exposed to 9.6 x 10^5^ PFU/ml of the marked viruses. Fewer than 2 marked viruses initiated all infections, and this was reflected in an average of 1.250 marked viruses in the midguts and 1.175 in the bodies (Fig 5B). When the mosquitoes were exposed to 1.06 x 10^6^ PFU/ml of marked viruses, a similar number were found with an average of 1.33 in the midguts, 1.155 in the bodies, and 1 in the legs/wings. However, no virus was found in the saliva of any of the mosquitoes (Fig 5E). While there is little or no difference in the titers seen between the two experiments, the 3x mutant showed consistent results and a more severe bottleneck than the wild-type virus (S5 Fig)

There was again a significant reduction in the number of marked viruses when the mosquitoes were exposed to 4.8x10^4^ PFU/ml of the 4x marked clones. Less than 2 clones initiated all infections, with an average of 1 marked virus in the midgut and 1.507 marked viruses in the bodies (Fig 5C). However, when exposed to 2.03 x10^6^ PFU/ml of virus, there was a limited amount of dissemination with a maximum of 3 marked clones per tissue (Fig 5F). Dissemination was observed in only two mosquitoes from this group; one mosquito showed dissemination to the legs/wings and one showed dissemination to the saliva. Interestingly, the legs/wings of the mosquito with saliva positive for virus did not show any evidence of infection. The results from this experiment were compared to the previous results in the wild-type and while there was a significant reduction in the number of marked clones in the bodies (S5 Fig), given the small number of mosquitoes that showed disseminated infection, it was not possible to determine statistical significance in the legs/wings and saliva compared to that previously seen. To determine if the titer was responsible for the number of clones, we calculated the titer using standard curves generated similar to those described in Forrester et al. (2012) [6]. These results can be found in S5A–S5F Fig). The samples could be divided into those with titers over 10^5^ pfu/ml and those under 10^5^ pfu/ml. However, there was no correlation between the number of marked viruses and the titer (S5G Fig) indicating that the number of marked viruses was not influenced by the titer of the infection.

### The effect of altered mutation frequencies on bottlenecks in the vertebrate host

**Fig 6:**
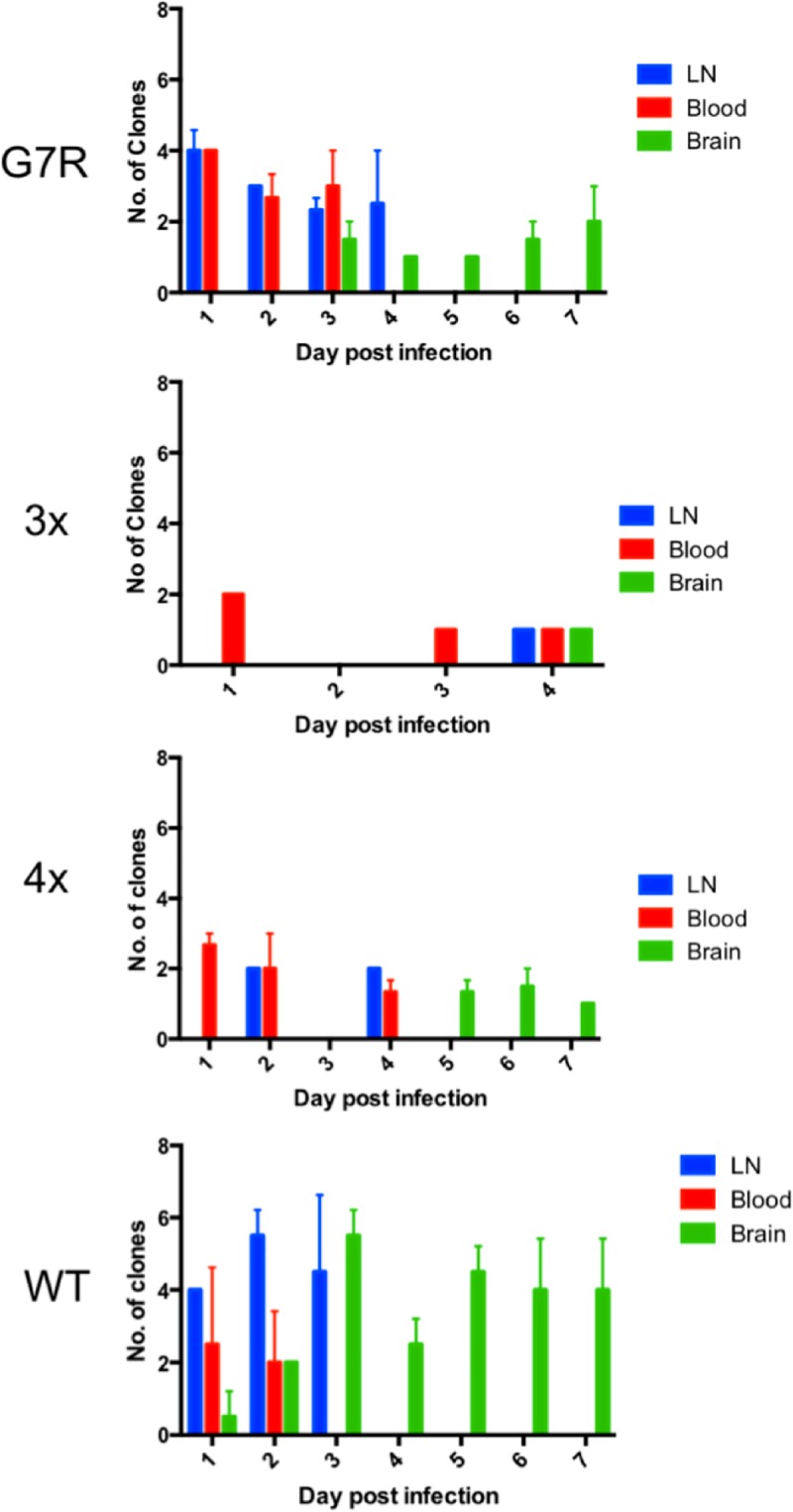
The number of marked clones present in the mouse in the lymph node (LN), blood and brain when infected with 103 PFU/ml of virus containing equal amounts of marked clones (WT, wild-type 68U201).

To investigate if the RdRp mutants were also attenuated in the vertebrate host, the marked viruses were injected into 6-week old CD-1 outbred mice and organs were collected daily for 7 days. The lymph nodes (LN), blood and brain were chosen for sampling because the LN is the primary site of replication, the blood is the means for transmission and the brain indicates the severity of the pathogenesis. Compared to the wild-type infection, there was a significant reduction in the number of marked viruses in the LN and the brain for all three RdRp mutants (Table 2 and Fig 6), whereas the number of marked viruses in the blood showed no significant change from the wild-type. In particular the 3x RdRp mutant showed a large reduction in the number of infected mice, with mice only being infected through day 4, though this could be due to random sampling of the animals and the nature of the terminal sampling carried out. There was no apparent bias in which clone was selected, and in the wild-type mice all 8 marked viruses were present in all organs. To confirm that this was not because of the presence of circulating blood in all the organs, a second infection was carried out where the mice were perfused to remove circulating virus in the blood, and all 8 clones were identified in the majority of tissues (S6 Fig).

**Table 2:** Statistical changes between wild-type and mutant viruses. Statistics were performed using two-way ANOVA, with post-hoc Tukey’s multiple comparisons tests. **<0.01, ****<0.0001 and ns–non-significant.

	LN	Blood	Brain
G7R	**	ns	****
3x	****	ns	****
4x	****	ns	****

## Discussion

Viral diversity is an important factor affecting RNA virulence and transmission. Generation of viral diversity allows the virus to effectively move between cells and cell types *in vivo*. It also increases the potential for the virus to continue its transmission cycle. Virus diversity is thought to be even more important for arboviruses, which infect a two-host system, as the virus must infect both a vertebrate host and a mosquito vector. It is well known that the virus undergoes numerous bottlenecks, or reductions in viral numbers, when going from host to host [6, 20]. This results in a small founding population that initiates infection within the new host. Bottlenecks imposed during transmission decrease the diversity of the viral population, therefore altering the course of infection by inhibiting the pathogen’s ability to adapt to the new host. The smaller the founding population, the slower adaption occurs [21]. A more diverse population covers more sequence space, meaning that the population is more likely to include virus variants required for efficient adaptation. Viruses that are able to produce more diversity with each replication event should be able to replenish diversity in the founding population quickly. Conversely, viruses that are hindered in their ability to generate diversity are hypothesized to be more sensitive to bottlenecks due to the inability to generate a diverse population from the founding population.

Previous work with fidelity mutants has demonstrated that they are attenuated *in vivo* [3, 13, 15, 22]. Low fidelity variants have also been linked to higher rates of recombination, causing more degenerate particles, which in turn stimulate type I interferon in the vertebrate host and RNA interference in the arthropod vector [23–27]. Specifically for VEEV, a recent study identified three mutations that decreased the fidelity of the VEEV vaccine strain TC-83 [11]. To test these mutants in a wild-type and unattenuated system, the same mutations were inserted into an enzootic strain of VEEV, 68U201. The hypothesis that these were fidelity mutants was tested, and the initial result suggested that the RdRp mutants encompassed both high and low fidelity tendencies. However, this result was not maintained upon serial passage, even though no reversion was observed in passage 5, in addition we did not see any other mutations that could act as pseudo-revertants. This suggested that other factors might be responsible for the attenuation of these mutant viruses.

As the phenotype of fidelity was not stable through 5 passages, other reasons for the attenuation were investigated. The data showed that there were changes in the mutation spectrum for each RdRp mutant, and given the recent work showing attenuation of Coxsackie B3 virus and Influenza A virus *in vivo* with 1-to-stop mutants [28], it was hypothesized that these altered individual mutation frequencies would result in an increased number of stop codons. Using an *in silico* approach, a computer model was used to determine if there was a change from the wild-type in the number of stop codons. All RdRp mutants showed a reduction in the number of predicted stop codons. Given that nascent genomes with additional stop codons are likely targeted for destruction, the absence of stop codons as a result of specific mutations likely results in a larger number of less-fit genomes (still containing critical 5’ and 3’ ends). Thus, replication in the G7R, 3x and 4x mutants results in more genomes that contain mutations that result in lower fitness, that if a stop codon had been inserted would otherwise be purified out during translation or packaging. From our data, it appears that attenuation of these viruses is a result of changing the frequency of specific mutations as a result of incorporating the specific RdRp mutants. Further evidence for this can be found in the reduced SI compared to the wild-type for the RdRp mutants, suggesting that there is an increase in the number of less fit genomes produced. Given the large discrepancy in the SI it is unlikely that these stop codons are the only mechanism leading to attenuation, but it is more likely a combination of factors. However, this work does raise interesting possibilities about the evolutionary pressures for specific mutation frequencies in viruses. This intriguing hypothesis will need to be evaluated experimentally both *in vivo* and *in vitro*.

When *Cx*. *taeniopus* mosquitoes were infected with the RdRp mutants as well as the wild-type virus, there was no significant decrease in the percentage of mosquitoes positive for virus in the body between the wild-type and mutant virus infections. However, there was a significant decrease in the legs/wings, and saliva. This indicates that changes in mutation frequency decrease dissemination within the mosquito vector. Only at the higher dose was virus detected in the legs/wings or saliva, and mosquitoes exposed to a higher dose also contained more clones than mosquitoes receiving a lower dose. When combined, these results support the hypothesis that any alteration in the generation of viral diversity results in an impaired ability to overcome bottlenecks imposed during dissemination through the mosquito compared to the wild-type virus. Although, it must be stated that some samples from the legs/wings and bodies may have been below the limit of detection, 10^1^ PFU, for real-time analysis.

This attenuation was recapitulated *in vivo* as mice infected with mutant viruses demonstrated increased time to death and increased survival rates compared to the wild-type virus 68U201. Interestingly, some mice infected with the attenuated mutants lost as much as 30% of their body weight but were able to recover. A possible explanation for this is that host immune systems are able to respond more effectively to these attenuated viruses once infection has been established, something not seen in the wild-type infection. This is not the first time this has been observed, as evidence for an improved immune response has been reported for low fidelity variants. The presence of lower fidelity resulted in an increased generation of degenerate virus particles that stimulate RIG-I, MDA5 and dendritic cell maturation [23–27, 29], resulting in a more effective immune response. When marked viruses with altered fidelity RdRps were injected subcutaneously into CD-1 mice there was a significant decrease in the number of clones isolated from the LN and the brain, but no change was observed in the blood. This suggests that the attenuation is likely due to the inability of the virus to spread between different cell or tissue types within the vertebrate host. Differences in infection between the different RdRp mutants were also observed. Notably, no 3x mutants were isolated after 4 days post infection, suggesting this attenuation contributed towards an inability to traverse the blood-brain-barrier. The lack of virus detected in the lymph node after day 4 for the G7R and 4x mutants, also suggests that these altered fidelity viruses are cleared from the organ, unlike the wild-type virus. RdRp mutants were only able to be isolated from the brain at later time points compared, to the wild-type. This delay in reaching the brain is evidence of the mutant virus population’s attenuation, which could potentially give the host immune response enough time to combat the infection. This would also account for the increased survival seen in these animals. The delayed spread of the RdRp mutants to the brain could also be due to its reduced ability to go through bottlenecks encountered during dissemination.

This work has demonstrated that the RdRp mutants described here have a reduced ability to traverse bottlenecks during infection of vertebrate hosts and mosquito vectors. There was a tentative association showing that the G7R RdRp mutant, which is a putative low fidelity phenotype, was less sensitive to bottlenecks compared to the 3x and 4x RdRp mutants, which showed putative high fidelity phenotype. However, all mutants were more sensitive to bottlenecks than the wild-type counterpart. This study has demonstrated that there are significant costs to altering the finely tuned balance of viral diversity, which has usually been conceived as a random accumulation of mutations [1, 15, 30]. Any perturbation of this either by increasing or decreasing fidelity, or altering the RdRp propensity for specific mutations may result in attenuation of the virus. Of great interest, the mutations that were inserted into the RdRp theoretically result in a reduction in the number of stop codons produced in the protein sequences due to altering the frequency of specific mutations. This suggests that VEEV and potentially other RNA viruses use the generation of stop codons as a mechanism for reducing the number of unfit genomes present in the mutant swarm, and have therefore evolved to optimize their genome to account for specific base mutation frequencies. If a higher percentage of mutations are more likely to generate a stop codon, they are less likely to encode signals and use resources to be either translated or packaged. This leads to the hypothesis that the presence of stop codons may be a method by which the virus reduces the number of less fit genomes produced. Regardless of whether these viruses are attenuated by changes in fidelity of the polymerase or as a result of reduced stop codon production, it is clear that any disruption of the mutation spectrum results in attenuation of the virus.

## Methods

### Ethics statement

This study was carried out in strict accordance with the recommendations in the Guide for the Care and Use of Laboratory Animals of the National Institutes of Health. The protocols (0209068 and 1309038) were approved by the Institutional Animal Care and Use Committee of the University of Texas Medical Branch.

### Cell culture and viruses

African green monkey kidney (Vero), Baby Hamster Kidney (BHK), and human lung fibroblast (MRC-5) cells were obtained from the American Type Culture Collection (Bethesda, MD) and maintained in Dulbecco’s minimal essential medium (DMEM) supplemented with 10% fetal bovine serum (FBS), and gentamycin (100 U/ml) in a 37°C, 5% CO2 incubator. U4.4, cells were maintained in Mitsuhashi and Maramorosch media supplemented with 20% FBS, 2% Sodium Bicarbonate (7.5%) and gentamycin (100 U/ml) in a 30°C CO2 incubator. Viruses were rescued from the enzootic subtype IE VEEV strain 68U201 as described previously and without further passage [31]. The wild type virus (genomic sequence in GenBank accession no. U34999) was isolated from one of the endemic foci in Guatemala in 1968 from a sentinel hamster. The isolated virus was passaged once in infant mice and twice in BHK cells before being cloned.

### Generation of genetically marked clones

Four mutations identified to cause altered fidelity in the VEEV vaccine strain TC-83 were cloned into the 68U201 backbone (Fig 1A; [11]. Mutations were inserted using joining PCR and the sequence of each RdRp mutant was verified prior to rescue of the virus. Following rescue of the RdRp mutants, 8 marked clones described in Forrester et al. 2012 were cloned into each of the RdRp mutant backbones.

All clones were rescued in BHK cells as previously described [32] and titers were determined by standard plaque assay on Vero cells [33]. To determine changes in fidelity, viruses were passaged once in Vero, MRC-5, and U4.4 cells. Cells were trypsinised (Vero and MRC-5) or scraped (U4.4) from the flask surface and then counted and resuspended in media. Viruses were then added to the cell suspension at a multiplicity of infection (MOI) of 0.1. Following one hour of incubation, with regular mixing, the cells were pelleted and resuspended in phosphate buffered saline (PBS) (Gibco, Carlsbad, CA). This was repeated three times and the final resuspension of the cells occurred in media. The cells were then plated out onto 24-well plates, with 1ml of cell suspension per well. Virus was harvested at 24 hours post infection and titered by standard plaque assay.

To ensure that the inserted mutations did not interfere with viral replication, standard replication curves were conducted. Cells were infected at a MOI of 10 and 0.01 in Vero cells in 24-well plates and incubated for one hour before being washed twice with PBS and overlaid with 2 ml of DMEM supplemented with 2% FBS and gentamycin (100U/ml). Virus was harvested by removing the entirety of the medium. Harvested virus was supplemented with FBS to a final concentration of 20%, and clarified by centrifugation at 3000rpm for 5 min. The supernatant was then removed and stored at -80°C. Viral titers were determined by standard plaque assays (Fig 1B).

Rescued viruses were passaged 5 times on Vero cells to ensure genetic stability. Viruses were harvested 2 days post-infection and diluted for a MOI of 0.01 before infection of Vero cells in the subsequent passage. Following passage 5, viral suspensions from passage 1 and 5 were placed into TRIzol (Invitrogen, Carlsbad, CA) and RNA extracted using the ZR Viral RNA Kit (Zymo Research, Irvine, CA) as per the manufacturer’s protocol. cDNA was produced using the Superscript III First Round Synthesis kit (Invitrogen, Carlsbad, CA) following the manufacturer's instructions. cDNA was amplified for sequencing using the Phusion enzyme (NEB, Madison, WI) in a 50ml volume following the manufacturer's instructions using primer sets that covered the entire genome. Products were sequenced on the ABI 3700 sequencer (ABI, 3500) to confirm the sequence of the passaged virus. RNA extractions were also sent for RNA sequencing (see below).

Rescued viruses from passage 1 and 5 were tested for susceptibility to 5’ fluorouracil in triplicate. Cells were pretreated with 0, 10, 25, 50, 100, 200, or 300 μg/ml of 5’ fluorouracil for 2 hours. The cells were then infected with 0.01 MOI of the RdRp mutants or wildtype 68U201. Medium containing the pretreatment amount of 5’ fluorouracil was added to the infected cells 1 hour post-infection. Virus was harvested 24 hours post-infection and titrated in duplicate using standard plaque assays.

Rescued viruses were used to test for specific infectivity of the virus. Standard plaque assays were carried out in triplicated as described above. RNA was also extracted using a QIAamp Viral RNA Mini kit according to the manufacturer’s protocol. Real-time RT-PCR was performed using primers against the nsP1 using primers (F 5’-TCACAGATAATGACCATGCTAACGC-3’, R 5’- TGTCTAGGATCGTATCGGATGGTTC-3’), and against the nsP3 using primers (F 5’- CTATTCCGCTTCTGTCCACTGGA-3’, R 5’- TTGTGTCCAATGCCGTTAACAGATG-3’). Real-time RT-PCR was carried out using iTaq Universal SYBR Green Mix (Bio-Rad) and iScript Reverse Transcriptase (Bio-Rad), with single steps of 10 min at 50°C and 3 min at 95°C, followed by cycling at 15 s at 95°C, and 30 s at 57°C for 45 cycles, and a melt curve from 55–95°C (+0.5°C/cycle).

### Mammalian infections

CD-1 mice (Charles Rivers, Wilmington, MA) were infected subcutaneously with 3 log_10_ PFU of virus. For survival studies, mice were bled on days 1, 2 and 3 to determine the level of viremia. The resulting blood samples were diluted 1:10 in DMEM supplemented with 10% FBS and stored at -80°C. Mice were weighed daily and monitored until they exhibited signs of paralysis, at which point they were sacrificed (Fig 2).

Mouse infections were also carried out using the set of marked clones. To determine the number of potential bottlenecks in a wild-type infection, mice were sampled daily (n = 3) following subcutaneous injection with 3 log_10_ PFU of virus. Each mouse was perfused using PBS and the brain, heart, lung, liver, spleen, kidney and lymph node were harvested. In a second round of experiments, mice were infected with the RdRp mutants that contained the marked clones, and were again sampled daily (n = 3). Each mouse was subjected to cardiac puncture to collect serum, and the brain and brachial lymph nodes were also harvested. All samples were tested for viral presence and titer by cytopathic effect (CPE) assay and standard plaque assay on Vero cells.

Mice used for oral mosquito exposure were injected via the tail vein with a mixture of all 8 clones in equal concentrations to generate an artificial viremia of known content. The mice were anaesthetized with ketamine/xylazine and then bled approximately 5 minutes post injection to estimate viremia titers before being presented to mosquitoes. Mosquitoes were allowed to feed on the mice for up to 45 minutes. Following a terminal blood draw, mice were sacrificed without gaining consciousness. Mouse manipulations were approved by the UTMB Institutional Animal Care and Use Committee.

### Mosquito infections

Cohorts of *C*. *taeniopus* mosquitoes (colony originating from Chiapas, Mexico) were sugar-starved for at least 16 hours, then allowed to feed for one hour on mice injected via the tail vein to generate viremia of predictable content, as described above. After one hour, engorged mosquitoes were incubated at 27°C and provided 10% sucrose *ad libitum*. At selected time points mosquitoes were chilled, their legs and wings removed, and then individuals were allowed to salivate for 45 minutes into a capillary tube containing FBS. The midguts were then dissected, and the remaining carcass was held separately. Midguts were cut in half and the residual blood was washed out using PBS. All mosquito tissues were placed into DMEM supplemented with 10% FBS, pen/strep and Fungizone (Sigma-Aldrich, St Louis, MO).

### Processing of tissues

All mosquito and vertebrate tissues were resuspended in DMEM supplemented with 10% FBS and gentamycin (for mosquito tissues Fungizone (Sigma-Aldrich)) was also added) and homogenized at 26 hz for 5 minutes, then subjected to centrifugation at 38206 x *g* for 10 minutes. Saliva samples were subjected to centrifugation at 6636 x *g* for 10 minutes prior to processing. All samples were tested for the presence of virus by CPE assay. Positive samples were stored at -80°C for subsequent analysis. For mosquito saliva samples, supernatants positive for CPE were used in a real-time RT-PCR assay, as the inconsistency in the amount of virus expectorated from the mosquito [34] would have resulted in some samples being below the limit of detection and thus the passaged supernatant was utilized [6].

### RNA extraction and RT-PCR methods

Virus suspensions were placed into TRIzol (Invitrogen, Carlsbad, CA) and RNA extracted using the ZR Viral RNA Kit (Zymo Research, Irvine, CA) as per the manufacturer’s protocol. Real time RT-PCR was carried out using the ABI 7900HT Fast Real-Time PCR system (ABI, Carlsbad, CA). Each reaction was performed using the TaqMan RNA-to-C_T_ 1-Step kit (ABI) as per the manufacturer’s instructions in a 10 μl reaction. The primers and probes were identical to those used in previously published work [6]. Each probe had a corresponding primer set that was designed to anneal flanking the polymorphic region of each variant. Every sample, run in duplicate, was tested for each variant. Positive and negative controls were run on each plate and all 8 clones were included as controls to ensure no cross-detection of the other clones by an individual probe. Additionally, serial dilutions with titers from 10^5^−10^1^ pfu/ml of the individual clones were used to create standard curves.

### Next generation sequencing and analysis

Viral RNA (0.05–1.7 mg) was fragmented by incubation at 94°C for 8 minutes in 19.5 μl of fragmentation buffer (Illumina, San Diego, CA). First and second strand synthesis, adapter ligation, and amplification of the library were performed using the Illumina TruSeq RNA Samplec Preparation kit as per the manufacturer’s protocol. Cluster formation of the library DNA templates was performed using the TruSeq PE Cluster Kit v3 (Illumina) and the Illumina cBot workstation as per the manufacturer’s protocol. Paired-end 50 base sequencing by synthesis was performed using TruSeq SBS kit v3 (Illumina) on an Illumina HiSeq 1500 as per the manufacturer’s protocol.

All reads were assembled using a pipeline previously described [7] and were assembled using the Venezuelan equine encephalitis virus strain 68U201 (GenBank accession #: U34999.1; [35]) as a reference sequence. Diversity was calculated using Shannon entropy [36] and a cut off of 1% was used for the analysis.

### Statistical analysis

Statistical analyses were carried out using GraphPad Prism and details are found in the text.

### Generating mutants in simulation

In order to quantify the extent to which point mutations create new stop codons, we simulated *in silico* the replication of (either 1m or 1bn) RNA strands for each variant, counting the number of individual strands produced that include new codons with either TAG, TAA, or TGA. For each variant, raw RNA data was obtained from the sequence of the infectious clone used to produce the virus. The algorithm begins at position 46 and reads each codon sequentially up to position 7538, skipping the opal stop at 5696. Reading continues at position 7546 and continues to 11338, this corresponds to the open reading frame for VEEV strain 68U201. Each base within each codon has a likelihood of mutation of 0.0001; if mutation occurs, the base changes randomly, according to the distribution given in Table 1. If a stop codon is produced at any point, this is recorded and the replicate counted as non-viable. The simulation code was written in C++ and is available for download at https://github.com/StanDeSiecle/Ihaventdonethisyet.

To determine the frequency of mutations leading to stop codons in the data generated from next generation sequencing of p1 viruses, each codon in the 68U201 genome was read sequentially. Using the substitution data from the p1 NGS data, we generated alternative codons if a substitution of any base was found to have occurred at any locus within that codon. We then recorded whether that substitution resulted in a stop codon. The proportion of stop codons to other mutations was then calculated for each virus and replicate.

## Supporting information

S1 Fig The changes in growth curves at an MOI of 0.01 and 10 for passage 1 and passage 5 viruses and the sensitivity to 5FU following passage 5 compared to passage 1. (TIFF)

S2 Fig The changes in mutation frequency for each individual cell type. The table shows the significance as determined by two-way ANOVA p<0.0001. Individual changes are found in the table. *p<0.05, **p<0.01, ***p<0.001, ****p<0.0001.
(TIFF)

S3 Fig Showing the numbers and graph of the mosquitoes assessed for dissemination on individual days. (TIFF)

S4 Fig Comparison of the fidelity mutants to the wild type for bodies. A). At 10^6 pfu/ml infection where the bodies were statistically different from the wild-type by two way ANOVA at P<0.0001, all fidelity clones showed differences from the wild-type by Dunnetts multiple comparisons test. B). At 10^5 pfu/ml infection where the bodies were statistically different by ANOVA p = 0.0077. However, by Dunnett’s multiple comparison test only the G14R showed significant difference from the wild-type at p<0.01.
(TIFF)

S5 Fig The relationship of titer to clone for A, B & C infections with low dose for G7R, 3x and 4x RdRp mutants, respectively. D, E & F, infections with high dose for G7R, 3x and 4x RdRp mutants, respectively. All titers were calculated from using standard curves run concurrently with the samples. G. The correlation between number of clones and titer for each of the samples for the G7R, 3x and 4x samples.
(TIFF)

S6 Fig The number of 68U201 marked clones present in the tissues of mice on days 1–7 post infection. Mice were infected with an equal mixture of 8 clones and perfused to remove infected blood before tissues were sampled.
(TIFF)

S1 Table The number of stop codons produced along the genome. The genome was sub-divided into 100 bins and the first column corresponds to the bin centers for that bin. This table was used to produce Fig 2C.
(DOCX)
