# Supplementary figures and images for: Viral RNA-dependent RNA polymerase mutants display an altered mutation spectrum resulting in attenuation in both mosquito and vertebrate hosts
Source: PLoS Pathog. 2019 Apr 4;15(4):e1007610. doi: 10.1371/journal.ppat.1007610 (PMC6467425; doi:10.1371/journal.ppat.1007610)

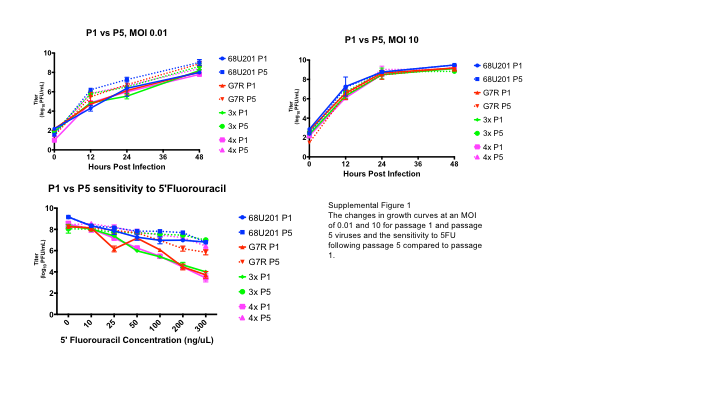

Supplement: S1 Fig — (TIFF) [file ppat.1007610.s001.tiff]

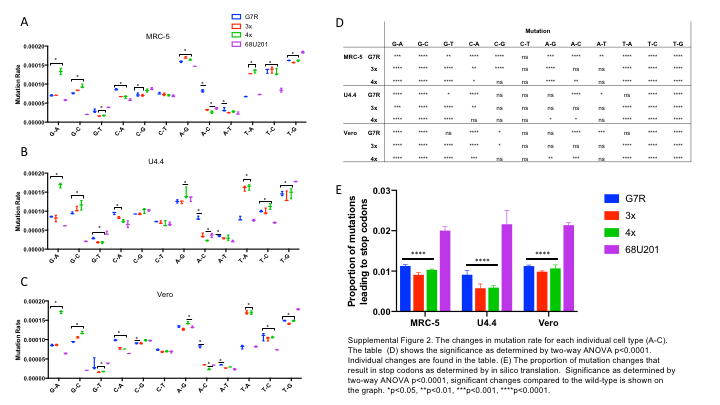

Supplement: S2 Fig — The table shows the significance as determined by two-way ANOVA p<0.0001. Individual changes are found in the table. *p<0.05, **p<0.01, ***p<0.001, ****p<0.0001. (TIFF) [file ppat.1007610.s002.tiff]

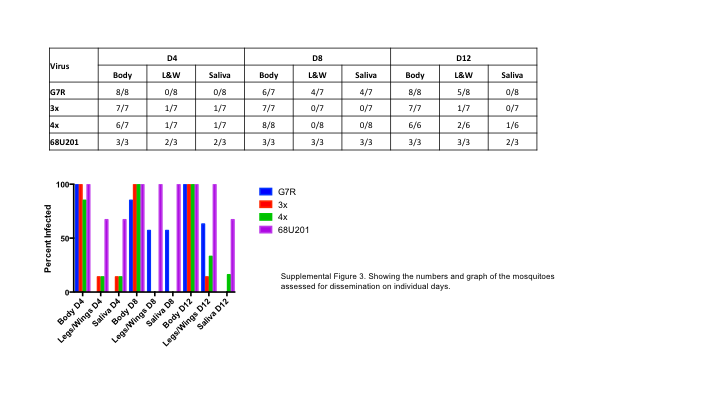

Supplement: S3 Fig — (TIFF) [file ppat.1007610.s003.tiff]

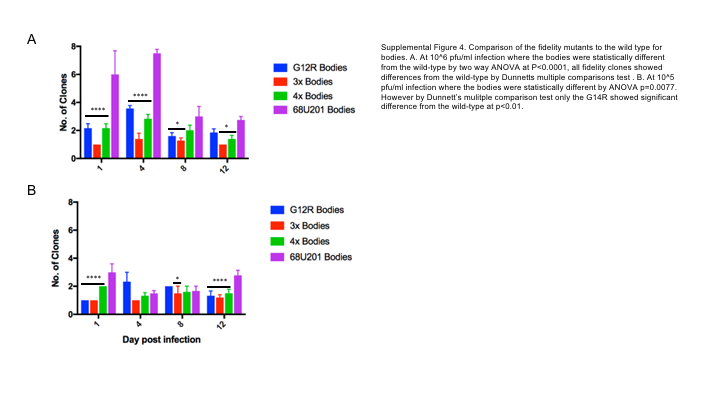

Supplement: S4 Fig — A). At 10^6 pfu/ml infection where the bodies were statistically different from the wild-type by two way ANOVA at P<0.0001, all fidelity clones showed differences from the wild-type by Dunnetts multiple comparisons test. B). At 10^5 pfu/ml infection where the bodies were statistically different by ANOVA p = 0.0077. However, by Dunnett’s multiple comparison test only the G14R showed significant difference from the wild-type at p<0.01. (TIFF) [file ppat.1007610.s004.tiff]

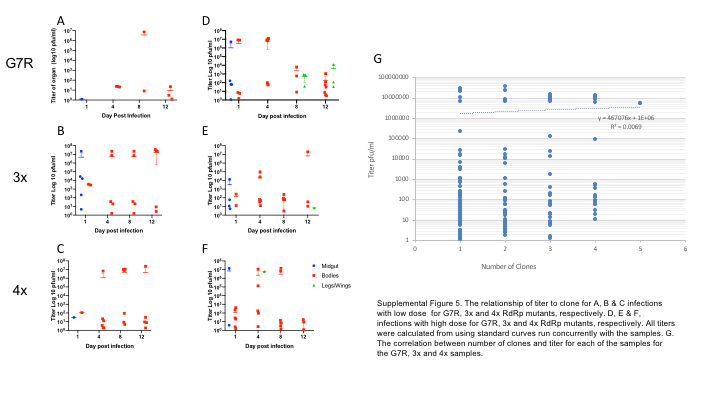

Supplement: S5 Fig — The relationship of titer to clone for A, B & C infections with low dose for G7R, 3x and 4x RdRp mutants, respectively. D, E & F, infections with high dose for G7R, 3x and 4x RdRp mutants, respectively. All titers were calculated from using standard curves run concurrently with the samples. G. The correlation between number of clones and titer for each of the samples for the G7R, 3x and 4x samples. (TIFF) [file ppat.1007610.s005.tiff]

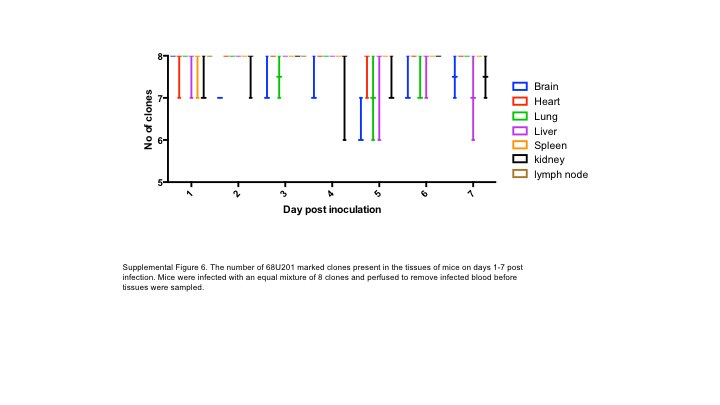

Supplement: S6 Fig — Mice were infected with an equal mixture of 8 clones and perfused to remove infected blood before tissues were sampled. (TIFF) [file ppat.1007610.s006.tiff]
