# Supplementary material for: Viral RNA-dependent RNA polymerase mutants display an altered mutation spectrum resulting in attenuation in both mosquito and vertebrate hosts
Source: PLoS Pathog. 2019 Apr 4;15(4):e1007610. doi: 10.1371/journal.ppat.1007610 (PMC6467425; doi:10.1371/journal.ppat.1007610)
Supplement: S1 Table — The genome was sub-divided into 100 bins and the first column corresponds to the bin centers for that bin. This table was used to produce Fig 2C. (DOCX) [file ppat.1007610.s007.docx]

**Supplemental Table 1.**

The number of stop codons produced along the genome. The genome was sub-divided into 100 bins and the first column corresponds to the bin centers for that bin. This table was used to produce figure 2C.

|  | **3x** | **4x** | **G7R** | **68U201** | **3x-diff** | **4x-diff** | **G7R-diff** |
| --- | --- | --- | --- | --- | --- | --- | --- |
| 96.769 | 227 | 240 | 272 | 365 | -138 | -125 | -93 |
| 215.23 | 302 | 322 | 332 | 377 | -75 | -55 | -45 |
| 328.05 | 440 | 443 | 415 | 424 | 16 | 19 | -9 |
| 440.87 | 330 | 295 | 330 | 439 | -109 | -144 | -109 |
| 553.69 | 372 | 432 | 426 | 460 | -88 | -28 | -34 |
| 666.51 | 497 | 570 | 521 | 595 | -98 | -25 | -74 |
| 779.33 | 465 | 469 | 447 | 524 | -59 | -55 | -77 |
| 892.15 | 406 | 404 | 426 | 398 | 8 | 6 | 28 |
| 1004.97 | 372 | 350 | 401 | 375 | -3 | -25 | 26 |
| 1117.79 | 207 | 199 | 159 | 209 | -2 | -10 | -50 |
| 1230.61 | 612 | 607 | 583 | 712 | -100 | -105 | -129 |
| 1343.43 | 400 | 440 | 355 | 402 | -2 | 38 | -47 |
| 1456.25 | 169 | 152 | 163 | 259 | -90 | -107 | -96 |
| 1569.07 | 255 | 249 | 288 | 444 | -189 | -195 | -156 |
| 1681.89 | 292 | 263 | 290 | 431 | -139 | -168 | -141 |
| 1794.71 | 316 | 320 | 408 | 434 | -118 | -114 | -26 |
| 1907.53 | 342 | 294 | 392 | 451 | -109 | -157 | -59 |
| 2020.35 | 548 | 557 | 553 | 660 | -112 | -103 | -107 |
| 2133.17 | 320 | 304 | 321 | 447 | -127 | -143 | -126 |
| 2245.99 | 222 | 223 | 295 | 283 | -61 | -60 | 12 |
| 2358.81 | 337 | 365 | 399 | 426 | -89 | -61 | -27 |
| 2471.63 | 272 | 281 | 283 | 239 | 33 | 42 | 44 |
| 2584.45 | 356 | 339 | 378 | 345 | 11 | -6 | 33 |
| 2697.27 | 298 | 358 | 312 | 380 | -82 | -22 | -68 |
| 2810.09 | 604 | 570 | 605 | 687 | -83 | -117 | -82 |
| 2922.91 | 334 | 364 | 345 | 414 | -80 | -50 | -69 |
| 3035.73 | 377 | 373 | 363 | 474 | -97 | -101 | -111 |
| 3148.55 | 401 | 402 | 393 | 445 | -44 | -43 | -52 |
| 3261.37 | 493 | 510 | 457 | 486 | 7 | 24 | -29 |
| 3374.19 | 511 | 558 | 543 | 521 | -10 | 37 | 22 |
| 3487.01 | 155 | 130 | 163 | 159 | -4 | -29 | 4 |
| 3599.83 | 304 | 298 | 267 | 383 | -79 | -85 | -116 |
| 3712.65 | 551 | 596 | 558 | 555 | -4 | 41 | 3 |
| 3825.47 | 309 | 279 | 274 | 352 | -43 | -73 | -78 |
| 3938.29 | 220 | 204 | 266 | 253 | -33 | -49 | 13 |
| 4051.11 | 273 | 271 | 317 | 358 | -85 | -87 | -41 |
| 4163.93 | 493 | 481 | 513 | 571 | -78 | -90 | -58 |
| 4276.75 | 263 | 223 | 280 | 351 | -88 | -128 | -71 |
| 4389.57 | 311 | 311 | 304 | 357 | -46 | -46 | -53 |
| 4502.39 | 385 | 418 | 426 | 566 | -181 | -148 | -140 |
| 4615.21 | 373 | 405 | 405 | 462 | -89 | -57 | -57 |
| 4728.03 | 392 | 420 | 389 | 465 | -73 | -45 | -76 |
| 4840.85 | 330 | 306 | 356 | 450 | -120 | -144 | -94 |
| 4953.67 | 459 | 432 | 430 | 416 | 43 | 16 | 14 |
| 5066.49 | 318 | 309 | 359 | 500 | -182 | -191 | -141 |
| 5179.31 | 225 | 241 | 231 | 333 | -108 | -92 | -102 |
| 5292.13 | 171 | 164 | 202 | 273 | -102 | -109 | -71 |
| 5404.95 | 205 | 219 | 219 | 216 | -11 | 3 | 3 |
| 5517.77 | 159 | 135 | 126 | 187 | -28 | -52 | -61 |
| 5630.59 | 330 | 291 | 319 | 379 | -49 | -88 | -60 |
| 5743.41 | 444 | 462 | 455 | 477 | -33 | -15 | -22 |
| 5856.23 | 539 | 580 | 606 | 650 | -111 | -70 | -44 |
| 5969.05 | 503 | 518 | 526 | 512 | -9 | 6 | 14 |
| 6081.87 | 402 | 401 | 399 | 422 | -20 | -21 | -23 |
| 6194.69 | 550 | 575 | 531 | 537 | 13 | 38 | -6 |
| 6307.51 | 216 | 220 | 225 | 247 | -31 | -27 | -22 |
| 6420.33 | 533 | 540 | 537 | 625 | -92 | -85 | -88 |
| 6533.15 | 460 | 487 | 426 | 476 | -16 | 11 | -50 |
| 6645.97 | 348 | 332 | 331 | 396 | -48 | -64 | -65 |
| 6758.79 | 330 | 370 | 304 | 411 | -81 | -41 | -107 |
| 6871.61 | 175 | 166 | 177 | 273 | -98 | -107 | -96 |
| 6984.43 | 264 | 251 | 231 | 278 | -14 | -27 | -47 |
| 7097.25 | 116 | 88 | 121 | 131 | -15 | -43 | -10 |
| 7210.07 | 429 | 459 | 400 | 427 | 2 | 32 | -27 |
| 7322.89 | 283 | 285 | 288 | 367 | -84 | -82 | -79 |
| 7435.71 | 269 | 325 | 341 | 446 | -177 | -121 | -105 |
| 7548.53 | 369 | 388 | 373 | 383 | -14 | 5 | -10 |
| 7661.35 | 309 | 322 | 312 | 332 | -23 | -10 | -20 |
| 7774.17 | 323 | 324 | 334 | 348 | -25 | -24 | -14 |
| 7886.99 | 304 | 342 | 316 | 346 | -42 | -4 | -30 |
| 7999.81 | 221 | 263 | 229 | 318 | -97 | -55 | -89 |
| 8112.63 | 529 | 513 | 520 | 590 | -61 | -77 | -70 |
| 8225.45 | 325 | 292 | 349 | 462 | -137 | -170 | -113 |
| 8338.27 | 335 | 389 | 343 | 516 | -181 | -127 | -173 |
| 8451.09 | 274 | 258 | 265 | 256 | 18 | 2 | 9 |
| 8563.91 | 451 | 480 | 493 | 559 | -108 | -79 | -66 |
| 8676.73 | 461 | 414 | 477 | 534 | -73 | -120 | -57 |
| 8789.55 | 501 | 500 | 485 | 566 | -65 | -66 | -81 |
| 8902.37 | 345 | 343 | 350 | 477 | -132 | -134 | -127 |
| 9015.19 | 403 | 368 | 417 | 533 | -130 | -165 | -116 |
| 9128.01 | 242 | 259 | 211 | 317 | -75 | -58 | -106 |
| 9240.83 | 712 | 765 | 786 | 718 | -6 | 47 | 68 |
| 9353.65 | 240 | 204 | 226 | 303 | -63 | -99 | -77 |
| 9466.47 | 155 | 153 | 171 | 217 | -62 | -64 | -46 |
| 9579.29 | 455 | 513 | 498 | 637 | -182 | -124 | -139 |
| 9692.11 | 607 | 610 | 644 | 594 | 13 | 16 | 50 |
| 9804.93 | 499 | 554 | 494 | 556 | -57 | -2 | -62 |
| 9917.75 | 380 | 490 | 359 | 413 | -33 | 77 | -54 |
| 10030.57 | 336 | 311 | 316 | 366 | -30 | -55 | -50 |
| 10143.39 | 370 | 372 | 377 | 407 | -37 | -35 | -30 |
| 10256.21 | 552 | 548 | 532 | 638 | -86 | -90 | -106 |
| 10369.03 | 473 | 491 | 550 | 557 | -84 | -66 | -7 |
| 10481.85 | 309 | 317 | 349 | 438 | -129 | -121 | -89 |
| 10594.67 | 419 | 398 | 438 | 552 | -133 | -154 | -114 |
| 10707.49 | 384 | 384 | 424 | 462 | -78 | -78 | -38 |
| 10820.31 | 262 | 235 | 277 | 280 | -18 | -45 | -3 |
| 10933.13 | 538 | 454 | 544 | 581 | -43 | -127 | -37 |
| 11045.95 | 316 | 322 | 295 | 392 | -76 | -70 | -97 |
| 11158.77 | 447 | 429 | 409 | 407 | 40 | 22 | 2 |
| 11271.59 | 647 | 754 | 690 | 749 | -102 | 5 | -59 |
